# Changes in acute kidney injury epidemiology in critically ill patients: a population-based cohort study in Korea

**DOI:** 10.1186/s13613-019-0534-7

**Published:** 2019-06-07

**Authors:** Subin Hwang, Hyejeong Park, Youngha Kim, Danbee Kang, Ho Suk Ku, Juhee Cho, Jung Eun Lee, Wooseong Huh, Eliseo Guallar, Gee Young Suh, Hye Ryoun Jang

**Affiliations:** 1 Department of Internal Medicine, Seoul Paik Hospital, Inje University School of Medicine, Seoul, Republic of Korea; 2 Center for Clinical Epidemiology, Samsung Medical Center, Sungkyunkwan University School of Medicine, Seoul, Republic of Korea; 3 Department of Clinical Research Design and Evaluation, Samsung Advanced Institute for Health Science and Technology, Sungkyunkwan University School of Medicine, Seoul, Republic of Korea; 4 Division of Nephrology, Department of Medicine, Samsung Medical Center, Sungkyunkwan University School of Medicine, 81 Irwon-ro, Gangnam-gu, Seoul, 06351 Republic of Korea; 5 Department of Epidemiology and Medicine and Welch Center for Prevention, Epidemiology and Clinical Research, Johns Hopkins Medical Institutions, Baltimore, MD USA; 6 Department of Critical Care Medicine, Samsung Medical Center, Sungkyunkwan University School of Medicine, 81 Irwon-ro, Gangnam-gu, Seoul, 06351 Republic of Korea

**Keywords:** Acute kidney injury, Critically ill patients, Intensive care unit, Mortality

## Abstract

**Background:**

Although no specific treatment facilitates renal tubular regeneration in acute kidney injury (AKI), the rapid increase in aging populations with more comorbidities and advances in critical care management are expected to change the epidemiology of AKI. However, few recent studies dissected the current epidemiologic characteristics of critically ill patients with AKI. We investigated recent epidemiologic changes in severe AKI in critically ill patients.

**Methods:**

All adult admissions to intensive care units (ICUs) in Korea from 2008 to 2015 were screened using the national health insurance review and assessment database, and 1,744,235 patients were included. Clinical characteristics and changes in AKI incidence and mortality rate were analyzed.

**Results:**

The incidence of AKI increased from 7.4% in 2008 to 8.3% in 2015 (*p* for trend < 0.001). Age-standardized AKI rate was 7018.6 per 100,000 person-years. In-hospital mortality significantly decreased from 39.1% in 2008 to 37.2% in 2015 (*p* for trend < 0.001) with 2427.6 deaths per 100,000 person-years. Patients with AKI showed higher in-hospital mortality, prolonged ICU length of stay, and higher total cost. Multivariable analysis showed increased risk of in-hospital mortality (adjusted odds ratio [OR] 3.74), mechanical ventilation (OR 2.87), ECMO (OR 6.99), and vasopressor requirement (OR 2.75) in patients with AKI.

**Conclusions:**

Recent advances in medical management for AKI have improved in-hospital mortality of critically ill patients with AKI despite increases in the elderly population and AKI incidence.

**Electronic supplementary material:**

The online version of this article (10.1186/s13613-019-0534-7) contains supplementary material, which is available to authorized users.

## Background

Acute kidney injury (AKI) is a common and serious problem in critically ill patients and poses a great burden on public health [1]. Although there is no specific treatment facilitating renal recovery or measures to counter the aging populations with more comorbidities, remarkable advances in critical care medicine may change the epidemiological characteristics of AKI in critically ill patients [2].

Previous studies on AKI incidence and mortality showed variable results depending on study design, selected cohort, and implementation of renal replacement therapy (RRT) [3–7]. A multinational prospective intensive care unit (ICU)-based study among 1802 patients reported that AKI severity was associated with mortality [8], and Kashani et al. [9] reported that AKI incidence remained relatively stable after adjusting for age and gender in one county in the USA between 2006 and 2014. However, few recent studies have dissected changes in the epidemiological characteristics of AKI in a large number of critically ill patients. Well-organized analyses of epidemiological changes of AKI such as incidence and mortality rates over time in large ICU populations and medical interventions may help optimize AKI public health policy and management.

In Korea, all citizens are required to join the National Health Security System as a mandatory social insurance program. Korean National Health Insurance (KNHI) is the key component of this system. In this study, the nationwide epidemiological characteristics of AKI in Korea were analyzed with data from the national Health Insurance Review and Assessment (HIRA) database provided by KNHI. Epidemiologic changes in incidence, mortality, and clinical characteristics of critically ill patients with AKI from 2008 to 2015 in Korea were investigated. We specifically focused on severe AKI that need to be recognized and managed more carefully and analyzed those underlying disease and management.

## Methods

### Data source and study population

We conducted a retrospective cohort analysis of the HIRA database from the Korean Ministry of Health. The database included virtually all ICU admissions in Korea during the study period.

ICU admissions were defined using claim codes AJ100-AJ590900, which are mandatory in HIRA for ICU management of in-hospital stays in all Korean hospitals. These codes are based on the Korean Classification of Diseases 6th edition (KCD-6), which is the modified version of the International Classification of Diseases 10th revision (ICD-10) adapted for the Korean health system [10]. All ICU stays during the same hospitalization were considered to be a single ICU admission. Similarly, hospital stays separated by < 2 days were considered to be the same hospital admission.

The study population comprised 1,744,235 patients aged 18 years and older who were admitted to the ICU for the first time and had no history of dialysis or AKI or ICU admission in the year before hospitalization from January 1, 2008 to December 31, 2015. AKI was defined when codes that identified an AKI (ICD-10 code N17) or RRT including continuous renal replacement therapy (CRRT; KNHI procedure codes O7031-O7035, and O7051-O7055), intermittent hemodialysis (IHD; O7020-O7021, O2011-O2012, O2081-O2083, and O9991), or peritoneal dialysis (PD; O7061-O7062, O7071-O7075, and E6581-E6593) were present.

Patients with a previous history of dialysis or AKI or ICU admission within 1 year were excluded using a combination of ICD-10 codes that identified an AKI, CRRT, IHD, or PD. We then excluded patients who received CRRT for other diseases/causes including mental and behavioral disorders, toxicity, or organ donors (*n* = 58,352). Patients who received CRRT for less than 3 days were also excluded because of the possibility of using CRRT for other diseases such as drug intoxication rather than AKI (*n* = 7794). Finally, a total of 1,678,089 patients were included in analysis.

### Measurement

ICD-10 codes were used to define comorbidities, procedures, prescriptions, and demographic characteristics [11]. Comorbidities were defined as reported codes during the year prior to admission and were defined and summarized as the Charlson index [12, 13], of which renal disease was defined by adding several disease codes to the Charlson comorbidities (Additional file 1: Table S1). Organ dysfunction was defined as the presence of the ICD-10 codes: respiratory (ICD-10 code J80, J960, R068, R092), cardiovascular (A419, E86, I951, I959, R570), hepatic (K720), hematologic (D65, D69), and neurologic (F23, F513, G931, G934, R402) [14].

Procedures such as mechanical ventilation (KNHI procedure codes; M5857, M5858, and M5860) and extracorporeal membrane oxygenation (ECMO; O1901-O1904, material codes; CAPIOX EBS CIRCUIT G5401008, QUADROX PLS G5501050, and CAPIOX EBS PMP CIRCUIT G5501008) were also analyzed depending on the presence of AKI. Use of vasopressor drugs such as dobutamine, dopamine, epinephrine, and norepinephrine for more than 2 days was identified using Korea drug and anatomical therapeutic chemical codes (codes; 148201BIJ, 38900BIJ, 148701BIJ, 148702BIJ, 429500BIJ, 152601BIJ, and 203101BIJ) [15].

Hospitals were classified according to capacity measured as the number of hospital beds and to specialties defined by Korean Health Law [16] as: hospital, a healthcare institution with more than 30 inpatient beds; general hospital, a hospital with more than 100 beds and more than seven specialty departments of internal medicine, surgery, pediatrics, obstetrics and gynecology, anesthesiology, pathology, and laboratory medicine; or tertiary hospital, a general hospital with more than 20 specialty departments that serves as a teaching hospital to medical students and nurses.

Study outcomes were in-hospital mortality, ICU and hospital length of stay, and total hospitalization cost. Hospital mortality was defined as receipt of insurance death codes. Total hospitalization cost including ICU stay was the amount of money reimbursed by KNHI to hospitals and patients for medical services endorsed by HIRA.

### Statistical analysis

Patients were divided into two groups by the presence of AKI during the hospitalization with ICU stay.

We obtained population estimates of Korean males and females for each year of age and calendar year from the Korean Statistical Information Service [17]. All analyses were conducted separately for males and females. Age-adjusted incidence rates of ICU hospitalizations and mortality were calculated by the direct method [18] using the Korean standard population from 2008 to 2015. To test for linear trends, we performed the Cochrane–Armitage trend test that included each category as a continuous variable in the regression models.

Mean and standard deviation (SD) or median and interquartile range were used to describe the distribution of continuous variables. *χ*^2^ test and Student’s *t* test were used to compare categorical and continuous variables, respectively.

Logistic regression models were used to identify univariable and multivariable predictors for severe AKI. Since patient survival could be clustered by hospital, we used hospital as a random intercept in mixed effects logistic model. Odds ratios (OR) with 95% confidence intervals (CI) were estimated using the model. We performed mixed effects logistic regression using the PROC GRLIMMIX procedures in SAS software.

Univariable mixed effects logistic regression analysis was used to identify possible risk factors for severe AKI. Next, the multivariable model only included variables that were found to be significant AKI risk factors on univariate analysis (*p *< 0.05). The final mixed effects logistic regression models included age, gender, tertiary hospital, year, Charlson index, comorbidities such as myocardial infarction (MI), congestive heart failure, cerebrovascular disease (CVD), rheumatologic disease, peptic ulcer disease, liver disease, diabetes mellitus, renal disease, cancer, acquired immune deficiency syndrome (AIDS)/human immunodeficiency virus (HIV), and organ dysfunction (respiratory, cardiovascular, hepatic, hematologic, neurologic). To evaluate the predictive accuracy of the risk prediction models, the area under receiver operating characteristics (AUROCs) was calculated.

A *p* value < 0.05 was considered significant for all analysis. Statistical analyses were performed using SAS and R visual analytics.

## Results

**Fig. 1:**
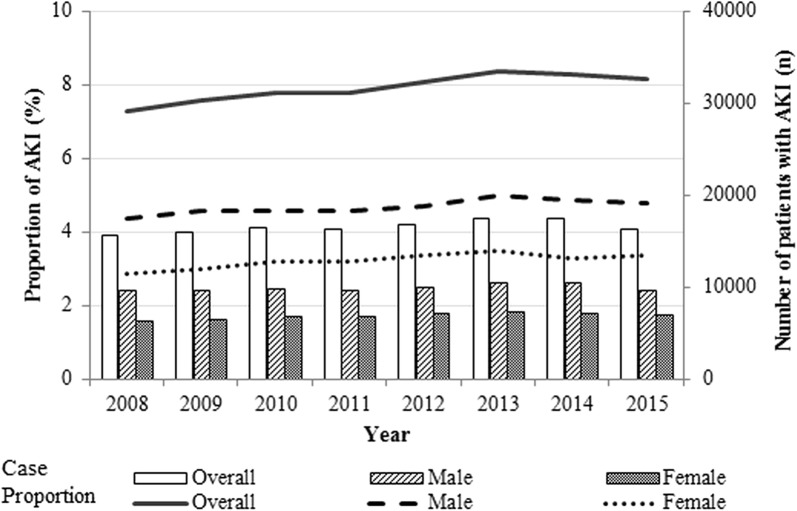
Yearly trends of acute kidney injury in critically ill patients in Korea between 2008 and 2015. Bars represent absolute number of critically ill patients with acute kidney injury (AKI); lines represent proportion of critically ill patients with AKI among all ICU patients. Incidence increased from 7.4% in 2008 to 8.3% in 2015 (*p* for trend < 0.001)

The overall incidence of AKI was 8.0%. The crude incidence of AKI increased significantly over time from 7.4% in 2008 to 8.3% in 2015 (*p* for trend < 0.001, Fig. 1). On average, the proportion of patients who received RRT was 20.4%, a slight decrease from 20.9% in 2008 to 19.2% in 2015. From 2008 to 2015, the mortality rate decreased in patients who received RRT (32.7–28.0%) and those who did not receive RRT (41.7–40.9%).

The mean (SD) age of all patients was 64.2 (15.5) years, and 57.4% were male. Patients with AKI were older, and had more comorbidities and organ dysfunction than patients without AKI (Table 1). Mechanical ventilation and vasopressors were used in 22.1% and 11.3% of all patients, respectively. Both mechanical ventilation and vasopressor drugs were more commonly used in patients with AKI compared to patients without AKI. Overall in-hospital mortality rate of ICU patients was 12.1%, and the in-hospital mortality of AKI patients was higher compared to patients without AKI (no AKI vs. AKI, 9.8% vs. 38.9%). Median ICU and hospital lengths of stay were also longer in patients with AKI.

**Table 1:** Characteristics of critically ill patients by the presence of acute kidney injury in Korea, 2008 to 2015

	Overall	No AKI	AKI	*p* value
Number of patients	1,678,089	1,544,968	133,121	
Gender				< 0.001^b^
Male	962,594 (57.4)	883,803 (57.2)	78,791 (59.2)	
Female	715,495 (42.6)	661,165 (42.8)	54,330 (40.8)	
Age, years	64.2 (15.5)	64 (15.5)	67.2 (15.2)	< 0.001^c^
Charlson index	2 (1–4)	2 (1–4)	3 (1–5)	< 0.001^d^
Comorbidity				
Myocardial infarction	95,571 (5.7)	87,606 (5.7)	7965 (6.0)	< 0.001^b^
Congestive heart failure	195,381 (11.6)	174,071 (11.3)	21,310 (16.0)	< 0.001^b^
Peripheral vascular disease	276,411 (16.5)	251,706 (16.3)	24,705 (18.6)	< 0.001^b^
Cerebrovascular disease	421,600 (25.1)	388,951 (25.2)	32,649 (24.5)	< 0.001^b^
Rheumatologic disease	81,210 (4.8)	73,570 (4.8)	7640 (5.7)	<0.001^b^
Peptic ulcer disease	517,857 (30.9)	475,376 (30.8)	42,481 (31.9)	< 0.001^b^
Liver disease	496,684 (29.6)	451,074 (29.2)	45,610 (34.3)	< 0.001^b^
Diabetes mellitus	570,759 (34.0)	513,760 (33.3)	56,999 (42.8)	< 0.001^b^
Renal disease	64,066 (3.8)	48,509 (3.1)	15,557 (11.7)	< 0.001^b^
Cancer	311,745 (18.6)	287,640 (18.6)	24,105 (18.1)	< 0.001^b^
AIDS/HIV	854 (0.1)	754 (0.1)	100 (0.1)	< 0.001^b^
Type of hospital				< 0.001^b^
Tertiary	659,767 (39.3)	607,539 (39.3)	52,228 (39.2)	
General	962,395 (57.4)	885,015 (57.3)	77,380 (58.1)	
Nursing care hospital	1089 (0.1)	963 (0.1)	126 (0.1)	
Other	54,838 (3.3)	51,451 (3.3)	3387 (2.5)	
Management procedures				
Mechanical ventilation	371,592 (22.1)	303,676 (19.7)	67,916 (51.0)	< 0.001^b^
ECMO	4618 (0.3)	2059 (0.1)	2559 (1.9)	< 0.001^b^
Vasopressor drugs	190,296 (11.3)	148,062 (9.6)	42,234 (31.7)	< 0.001^b^
Organ dysfunction				
Respiratory	68,061 (4.1)	49,839 (3.2)	18,222 (13.7)	< 0.001^b^
Cardiovascular	144,774 (8.6)	10,5992 (6.9)	38,782 (29.1)	< 0.001^b^
Hepatic	7475 (0.5)	5216 (0.3)	2259 (1.7)	< 0.001^b^
Hematologic	93,124 (5.6)	67,683 (4.4)	25,441 (19.1)	< 0.001^b^
Neurologic	38,466 (2.3)	31,913 (2.1)	6553 (4.9)	< 0.001^b^
Outcomes				
Hospital mortality	203,373 (12.1)	151,550 (9.8)	51,823 (38.9)	< 0.001^b^
ICU length of stay, days	3 (2–8)	3 (2–7)	8 (3–22)	< 0.001^d^
Hospital length of stay, days	13 (7–24)	13 (7–24)	17 (8–35)	< 0.001^d^
Total cost, USD^a^	5208 (2617–8814)	5111 (2566–8487)	7386 (3347–15,751)	< 0.001^d^

Values are numbers and proportions, except for age and Charlson index (mean and SD), ICU length of stay, hospital length of stay, and total cost (median and interquartile)

*AIDS* acquired immune deficiency syndrome, *AKI* acute kidney injury, *HIV* human immunodeficiency virus, *ECMO* extracorporeal membrane oxygenation, *ICU* intensive care unit, *USD* US dollars

^a^1 USD = 1158 Korean won (exchange rate as of December 1, 2015)

^b^Chi-squared test

^c^Student’s *t* test

^d^Wilcoxon–Mann–Whitney test

**Fig. 2:**
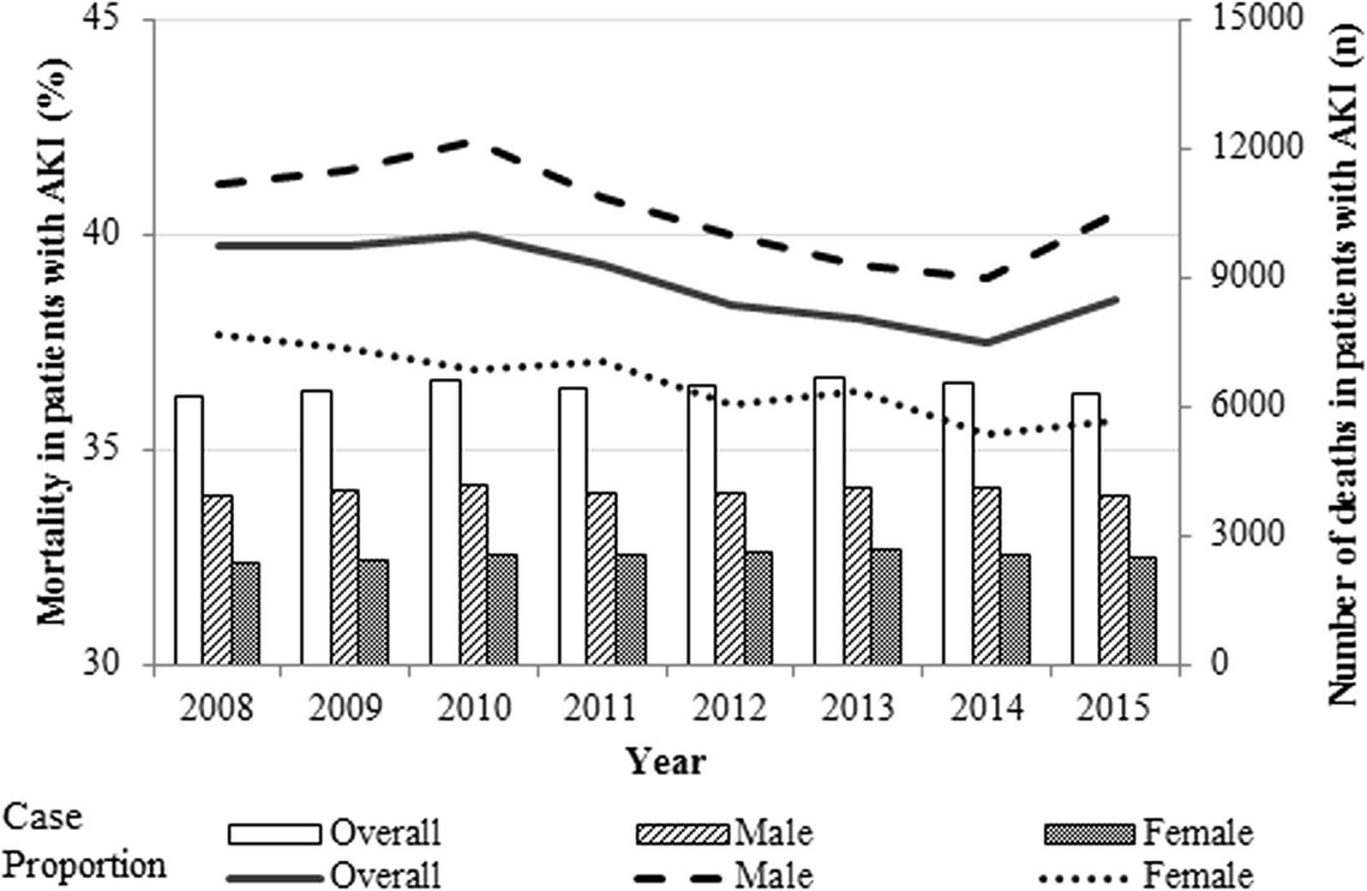
In-hospital mortality rate in critically ill patients with acute kidney injury in Korea between 2008 and 2015. Bars represent absolute number of deaths in critically ill patients with acute kidney injury (AKI); lines represent mortality of critically ill patients with AKI. Mortality decreased from 39.1 to 37.2% (*p* for trend < 0.001)

The peak number of critically ill patients with AKI was in the eighth decade of life: at age 73 years in males and age 78 years in females. From 2008 to 2015, the age-standardized incidence of AKI in critically ill patients was 7018.6 per 100,000 person-years (4571.9 per 100,000 person-years in males and 2446.6 per 100,000 person-years in females). The incidence of AKI was higher in males until the age of 80, but higher in females from age 80 and older (Additional file 2: Fig. S1). Mortality rates of critically ill patients with AKI decreased significantly over time from 39.1 to 37.2% (*p* for trend < 0.001, Fig. 2). There were 2427.6 deaths of critically ill patients with AKI among 100,000 Koreans per year (1605.6 deaths per 100,000 males and 822.0 deaths per 100,000 females; Additional file 3: Fig. S2).

Patients with AKI were more likely to be older (OR, 1.01; 95% CI, 1.01–1.01), male (OR, 1.02; 95% CI, 1.02–1.03), have admitted to the hospital recently (OR, 1.18; 95% CI, 1.17–1.20) and have more comorbidities of congestive heart failure (OR, 1.16; 95% CI, 1.14–1.18), diabetes mellitus (OR, 1.26; 95% CI, 1.25–1.28), and renal disease (OR, 3.45; 95% CI, 3.37–3.52). They also had more organ dysfunction associated with higher incidence of AKI compared to (*p *< 0.001, Table 2). Adjusted ORs for AKI were lower for the comorbidities of MI and CVD (OR, 0.85; 95% CI, 0.83–0.87 and OR, 0.78; 95% CI, 0.77–0.79). The area under the curve (AUC) value for predicted probabilities was 0.780 (95% CI, 0.779–0.782).

**Table 2:** Odds ratio (95% CI) for acute kidney injury in intensive care unit hospitalization in Korea, 2008 to 2015

	Crude odds ratio (95% CI)	*p* value^a^	Adjusted odds ratio (95% CI)	*p* value^a^
Age (continuous)	1.02 (1.02–1.02)	< .001	1.01 (1.01–1.01)	< .001
Gender, male	1.09 (1.08–1.10)	< .001	1.02 (1.02–1.03)	< .001
Tertiary hospital	0.94 (0.91–0.97)	< .001	1.05 (1.05–1.06)	0.365
Year	1.02 (1.02–1.02)	< .001	1.18 (1.17–1.20)	< .001
Charlson index	1.08 (1.08–1.09)	< .001	0.98 (0.95–1.02)	< .001
Comorbidity				
Myocardial infarction	1.00 (0.97–1.02)	0.857	0.85 (0.83–0.87)	< .001
Congestive heart failure	1.50 (1.48–1.53)	< .001	1.16 (1.14–1.18)	< .001
Peripheral vascular disease	1.12 (1.15–1.18)	< .001	0.94 (0.93–0.96)	< .001
Cerebrovascular disease	0.97 (0.95–0.98)	< .001	0.78 (0.77–0.79)	< .001
Rheumatologic disease	1.23 (1.20–1.26)	< .001	0.98 (0.96–1.01)	0.239
Peptic ulcer disease	1.07 (1.06–1.08)	< .001	0.88 (0.87–0.89)	< .001
Liver disease	1.28 (1.27–1.30)	< .001	0.99 (0.98–1.01)	0.431
Diabetes mellitus	1.61 (1.60–1.63)	< .001	1.26 (1.25–1.28)	< .001
Renal disease	4.23 (4.15–4.32)	< .001	3.45 (3.37–3.52)	< .001
Cancer	1.06 (1.05–1.08)	< .001	0.72 (0.70–0.74)	< .001
AIDS/HIV	1.46 (1.18–1.81)	< .001	0.85 (0.67–1.06)	0.151
Organ dysfunction				
Respiratory	4.84 (4.75–4.94)	< .001	2.97 (2.91–3.04)	< .001
Cardiovascular	5.89 (5.81–5.97)	< .001	4.24 (4.18–4.31)	< .001
Hepatic	4.56 (4.33–4.81)	< .001	3.46 (3.26–3.67)	< .001
Hematologic	5.24 (5.15–5.33)	< .001	3.64 (3.57–3.70)	< .001
Neurologic	2.49 (2.42–2.56)	< .001	1.84 (1.78–1.90)	< .001

Adjusted for age, gender, tertiary hospital, year, Charlson index, myocardial infarction, congestive heart failure, cerebrovascular disease, rheumatologic disease, peptic ulcer disease, liver disease, diabetes, renal disease, cancer, AIDS/HIV, and organ dysfunction (respiratory, cardiovascular, hepatic, hematologic, neurologic)

*AIDS* acquired immune deficiency syndrome, *CI* confidence interval, *HIV* human immunodeficiency virus

^a^Mixed effects logistic regression model

Hospital mortality was significantly higher in patients with AKI after adjusting for age, gender, tertiary hospital, year, Charlson index, comorbidities, and organ dysfunction (adjusted OR, 3.74; 95% CI, 3.68–3.79). The appliance rates of mechanical ventilation (adjusted OR, 2.87; 95% CI, 2.83–2.91), ECMO (adjusted OR, 6.99; 95% CI, 6.53–7.19), and vasopressors (adjusted OR, 2.75; 95% CI, 2.71–2.79) were also higher in patients with AKI (Table 3).

**Table 3:** Adjusted odds ratios (95% CI) of management procedures and outcomes associated with critically ill patients with acute kidney injury

	No. of patients	No. of AKI patients	Crude odds ratio (95% CI)	*p* value^a^	Adjusted odds ratio (95% CI)	*p* value^a^
In-hospital mortality	203,373	51,823	6.33 (6.25–6.42)	< 0.001	3.74 (3.68–3.79)	< 0.001
Mechanical ventilation	371,592	67,916	4.72 (4.66–4.77)	< 0.001	2.87 (2.83–2.91)	< 0.001
ECMO	4618	2559	16.18 (15.24–17.17)	< 0.001	6.99 (6.53–7.19)	< 0.001
Vasopressor drugs	181,936	40,523	4.71 (4.64–4.77)	< 0.001	2.75 (2.71–2.79)	< 0.001

Adjusted for age, gender, tertiary hospital, year, Charlson index, myocardial infarction, congestive heart failure, cerebrovascular disease, rheumatologic disease, peptic ulcer disease, liver disease, diabetes, renal disease, cancer, AIDS/HIV, and organ dysfunction (respiratory, cardiovascular, hepatic, hematologic, neurologic)

*AIDS* acquired immune deficiency syndrome, *AKI* acute kidney injury, *CI* confidence interval, *ECMO* extracorporeal membrane oxygenation, *HIV* human immunodeficiency virus

^a^Mixed effects logistic regression model

## Discussion

This study was a population-based, nationwide epidemiological analysis of recent trends in AKI in critically ill adult patients in Korea, a major Asian country with sufficient medical resources. The AKI incidence increased from 7.4% in 2008 to 8.3% in 2015. AKI was a consistently strong risk factor for in-hospital mortality after adjusting for age, gender, tertiary hospital, year, Charlson index, organ dysfunction, and several relevant comorbidities. AKI was also associated with the increased use of ECMO, mechanical ventilation, and vasopressors, resulting in longer ICU and hospital stays, as well as higher costs. However, overall mortality rates of critically ill patients with AKI significantly decreased from 39.1 to 37.2% during 8 recent years in Korea.

The overall incidence of AKI in critically ill patients significantly increased during the study period. An epidemiological study conducted at the Mayo Clinic in a population-based cohort found no significant changes in the temporal trend over the last decade of sniffer-diagnosed AKI incidence [9]. AKI incidence tended to increase in several large cohort epidemiological studies in ICU settings [19–21]. However, these studies had limitations in representability or generalization since most included patients from limited regions or for short periods. Our study strongly supports these previous studies by analyzing data from a nationwide, continuously well-collected database. The increasing incidence of AKI in ICU patients may be attributed to several factors including increased utilization of medical resources, introduction of new drugs with potential nephrotoxicity, an increasing aging population, and increases in chronic kidney disease.

Most underlying diseases and serious comorbidities of renal disease, congestive heart failure, liver disease, diabetes mellitus, cancer, and AIDS/HIV increased AKI risk. However, MI and CVD did not increase AKI risk. Overall outcomes for MI and CVD have improved due to advances in diagnostic techniques and standardized treatment including revascularization within the golden time. Although MI and CVD were reported to be associated with contrast-induced nephropathy (CIN) [22, 23], these comorbidities were associated with lower risk of AKI in our study. Our findings were consistent with previous studies reporting that interventions improve patient outcome without increasing AKI risk [24, 25]. Our results might reflect favorable CIN outcomes because CIN prophylaxis protocols have been widely applied in most Korean hospitals [26, 27]. This result suggests that early recognition of high-risk patients and adequate management with reno-protective protocols may prevent AKI.

A study in England reported that in-hospital fatality of AKI was unchanged at around 30% in 2003–2008 but increased to 41% in 2008–2013 [28]. In Canada, Wald et al. [21] showed an increase in AKI incidence between 1996 and 2010, while 90-day mortality significantly declined. However, the two populations in these national epidemiologic studies included only ICU patients requiring RRT. Our key finding was a significant decline in mortality from 2008 to 2015 in virtually all critically ill Korean patients with AKI regardless of RRT. ICU patients with AKI requiring RRT may not represent the entire spectrum of critically ill patients with AKI. Therefore, our study more adequately showed epidemiologic changes in these patients compared to previous studies. Recent medical and technological advances in RRT and circulatory or extracorporeal life support systems may have improved the outcomes of critically ill patients with AKI. Our results are supported by previous studies reporting that crude mortality decreased from 1996 to 2005 among all admissions for adults in 20 Australian ICUs [19], and the mortality of AKI patients who did not require RRT decreased from 1998 to 2013 [29].

The past decade has seen remarkable advances in clinical practice of critical care medicine. In particular, ECMO for critically ill patients with the most severe respiratory or cardiac failure has advanced and been actively applied [30]. We found that mechanical ventilation, vasopressor drugs, and ECMO were more commonly used in critically ill patients with AKI, reflecting the higher severity of the patients. Although variables such as Sequential Organ Failure Assessment (SOFA)/Acute Physiology and Chronic Health Evaluation II (APACHE II) score may reflect the severity of patients more accurately, these variables were not available due to the nature of reimbursement data. To minimize this limitation, the Charlson comorbidity index was used to represent patient severity of illness and for risk adjustment. The Charlson index has been reported as an alternative method of risk adjustment facilitating comparisons between ICUs [31]. The necessity of these management procedures significantly increased in critically ill patients with AKI after adjusting for age, gender, and comorbidities. These data were supported by prior studies, showing that AKI may substantially contribute to prolonged hospitalization and increase in medical costs [1, 20, 32].

This study had several limitations. The first was the lack of detailed clinical data such as serum creatinine or urine output because our study was based on a national registry database [33]. We did not use a contemporary consensus definition of AKI and could not distinguish AKI severity. In contrast to most ICU cohort studies reporting the incidence of AKI of 40–60%, a relatively lower incidence (less than 10%) of AKI was found in our study. Selection bias cannot be excluded, but the incidence of severe AKI is comparable with other studies. In a previous study using AKIN and KDIGO criteria, stage 3 AKI was reported to be about 7% [34]. In a recently published study using also NHI data, the incidence of AKI in patients who were admitted to the ICU and received CRRT was 3.8% in Korea [35]. The incidence of AKI in these studies was similar to our study. The primary purpose of our study was to evaluate the epidemiologic trends and mortality rates of severe AKI, therefore we believe that the incidence of AKI was sufficient to support our study aim. The second limitation was that we did not include the cause of AKI or specific types or stage of chronic kidney disease as underlying renal disease in analyses. However, we believe that for the primary purpose of study, we sufficiently analyzed well-collected reliable data from a large cohort without bias from researchers or medical institutions. The NHIS database was not collected for research purposes and therefore could be biased [36]. In Korea, the government provides special insurance benefits to procedure and treatment related to the disease based on disease codes. Therefore, as the Department of NHIS routinely audits the claims, such data are considered reliable and have been used in numerous peer-reviewed publications. A comparison of the medical record reports with the diagnostic results of the HIRA data showed that 75.9% of the diagnoses in the hospital were consistent with the diagnostic results of the medical records [37] and another validation study of discharge diagnoses in the NHIS database compared against medical records found an overall positive predictive value of 83.4% [38]. Additionally, generalization of our results might be difficult because of the inherent nature of the epidemiologic database in Korea. However, the main results of this study may reflect recent changes in AKI epidemiology because the Korean medical system and facilities are comparable with those in Western countries.

Despite these limitations, the major strength of this study was analyses of the current trends in incidence and mortality of critically ill patients with AKI in virtually all ICU admissions in Korea for 8 years. Confounding factors including variability in diagnosis and treatment influenced by medical costs might be minimized because of the mandatory medical insurance system and reimbursement system of the Korean government. An additional strength was that we analyzed the impact of AKI on important ways to manage critically ill patients such as vasopressor use, mechanical ventilation, and ECMO.

## Conclusions

This study of a large, population-based cohort admitted to Korean ICUs over the past decade demonstrated that overall mortality significantly decreased in critically ill patients with AKI despite the increased incidence of AKI. Further studies investigating specific treatment strategies for improving renal outcomes in critically ill patients with AKI are required.

## Additional files

**Additional file 1: Table S1.** ICD-10 coding algorithms for comorbidities based on Charlson comorbidities.

**Additional file 2: Fig. S1.** Number of patients with acute kidney injury and age-standardized rate by age and gender in Korea between 2008 and 2015. The peak number of critically ill patients with acute kidney injury (AKI) was found at age 73 years in males and 78 years in females. AKI incidence was higher in males until age 80, but higher in females from age 80 and older.

**Additional file 3: Fig S2.** Number of deaths in critically ill patients with acute kidney injury and age-standardized in-hospital mortality by age and gender in Korea between 2008 and 2015. In-hospital mortality showed an upward trend by age. The increase was more rapid in females than in males after age 70.

## Data Availability

Not applicable.

## Abbreviations

AKI: acute kidney injury
ICU: intensive care unit
ECMO: extracorporeal membrane oxygenation
RRT: renal replacement therapy
KNHI: Korean National Health Insurance
HIRA: Health Insurance Review and Assessment
KCD: Korean Classification of Diseases
ICD: International Classification of Diseases
IHD: intermittent hemodialysis
PD: peritoneal dialysis
MI: myocardial infarction
CVD: cerebrovascular disease
AIDS/HIV: acquired immune deficiency syndrome/human immunodeficiency virus
CIN: contrast-induced nephropathy
