# Supplementary material for: Changes in acute kidney injury epidemiology in critically ill patients: a population-based cohort study in Korea
Source: Ann Intensive Care. 2019 Jun 7;9:65. doi: 10.1186/s13613-019-0534-7 (PMC6555834; doi:10.1186/s13613-019-0534-7)
Supplement: Supplementary file 1 — Additional file 1: Table S1. ICD-10 coding algorithms for comorbidities based on Charlson comorbidities. [file 13613_2019_534_MOESM1_ESM.docx]

**Additional file 1: Table S1. ICD-10 coding algorithms for comorbidities based on Charlson comorbidities**.

| **Charlson comorbidity index** | **ICD-10** |
| --- | --- |
| Myocardial infarction | I21, I22, I252 |
| Congestive heart failure | I099, I110, I130, I132, I255, I420, I425, I426, I427, I428, I429, I43, I50, P290 |
| Peripheral vascular disease | I70, I71, I731, I738, I739, I771, I790, I792, K551, K558, K559, Z958, Z959 |
| Cerebrovascular disease | G45, G46, I60, I61, I62, I63, I64, I65, I66, I67, I68, I69, H340 |
| Dementia | F00, F01, F02, F03, G30, F051, G311 |
| Chronic pulmonary disease | I278, I279, J40, J41, J42, J43, J44, J45, J46, J47, J60, J61, J62, J63, J64, J65, J66, J67, J684, J701, J703 |
| Connective tissue disease | M05, M06, M315, M32, M33, M34, M351, M353, M360 |
| Peptic ulcer disease | K25, K26, K27, K28 |
| Mild liver disease | B18, K700, K701, K702, K703, K709, K713, K714, K715, K717, K73, K74, K760, K762, K763, K764, K768, K769, Z944 |
| Moderate or severe liver disease (3) | I850, I859, I864, I982, K704, K711, K721, K729, K765, K766, K767 |
| Diabetes without complications | E100, E101, E106, E108, E109, E110, E111, E116, E118, E119, E120, E121, E126, E128, E129, E130, E131, E136, E138, E139, E140, E141, E146, E148, E149 |
| Diabetes with complications (2) | E102, E103, E104, E105, E107, E112, E113, E114, E115, E117, E122, E123, E124, E125, E127, E132, E133, E134, E135, E137, E142, E143, E144, E145, E147 |
| Paraplegia and hemiplegia (2) | G041, G114, G800, G81, G82, G830, G831, G832, G833, G834, G839 |
| Renal disease (2) | I120, I131, N030, N031, N032, N033, N034, N035, N036, N037, N038, N039, N050, N051, N052, N053, N054, N055, N056, N057, N058, N059, N18, N19, N250, Z490, Z491, Z492, Z940, Z992 |
| Cancer (2) | C00, C01, C02, C03, C04, C05, C06, C07, C08, C09, C10, C11, C12, C13, C14, C15, C16, C17, C18, C19, C20, C21, C22, C23, C24, C25, C26, C30, C31, C32, C33, C34, C37, C38, C39, C40, C41, C43, C45, C46, C47, C48, C49, C50, C51, C52, C53, C54, C55, C56, C57, C58, C60, C61, C62, C63, C64, C65, C66, C67, C68, C69, C70, C71, C72, C73, C74, C75, C76, C81, C82, C83, C84, C85, C88, C90, C91, C92, C93, C94, C95, C96, C97 |
| Metastatic carcinoma (6) | C77, C78, C79, C80 |
| AIDS/HIV (6) | B20, B21, B22, B24 |

*ICD* international statistical classification of disease
