# Supplementary figures and images for: Changes in acute kidney injury epidemiology in critically ill patients: a population-based cohort study in Korea
Source: Ann Intensive Care. 2019 Jun 7;9:65. doi: 10.1186/s13613-019-0534-7 (PMC6555834; doi:10.1186/s13613-019-0534-7)

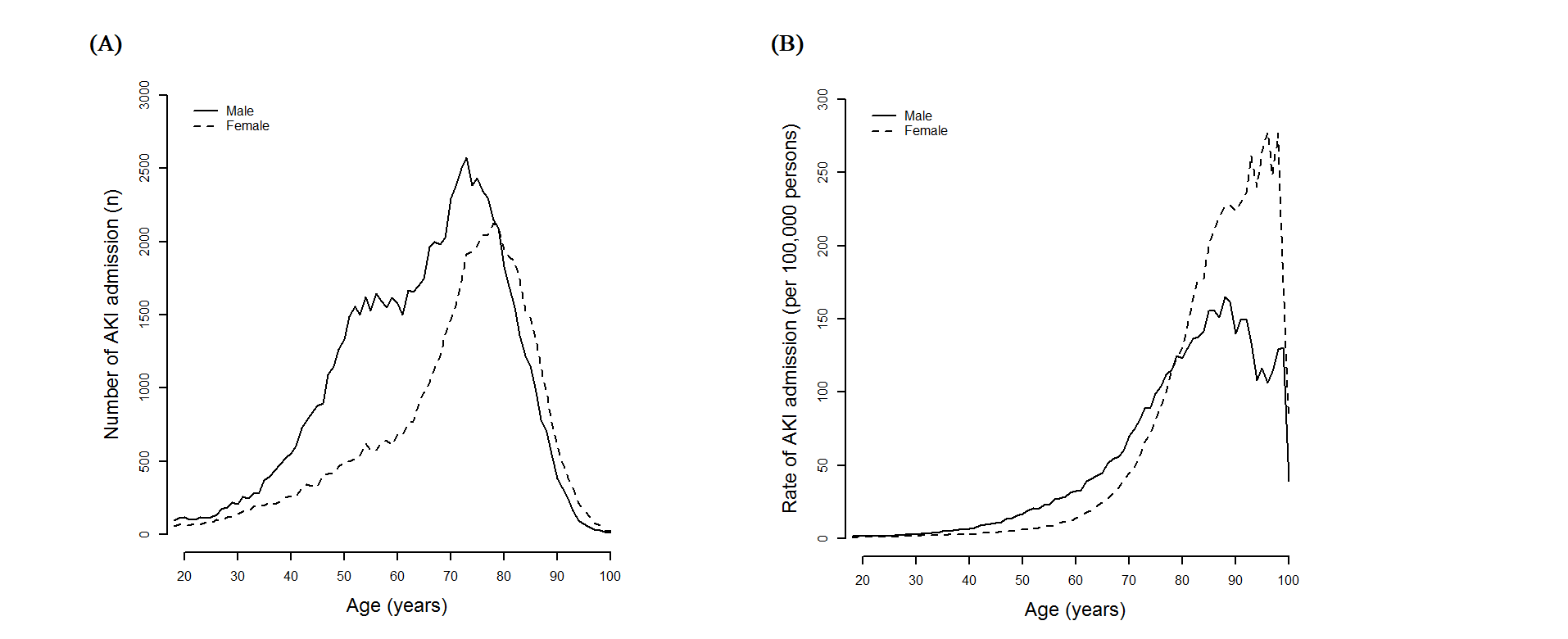

Supplement: Supplementary file 2 — Additional file 2: Fig. S1. Number of patients with acute kidney injury and age-standardized rate by age and gender in Korea between 2008 and 2015. The peak number of critically ill patients with acute kidney injury (AKI) was found at age 73 years in males and 78 years in females. AKI incidence was higher in males until age 80, but higher in females from age 80 and older. [file 13613_2019_534_MOESM2_ESM.tif]

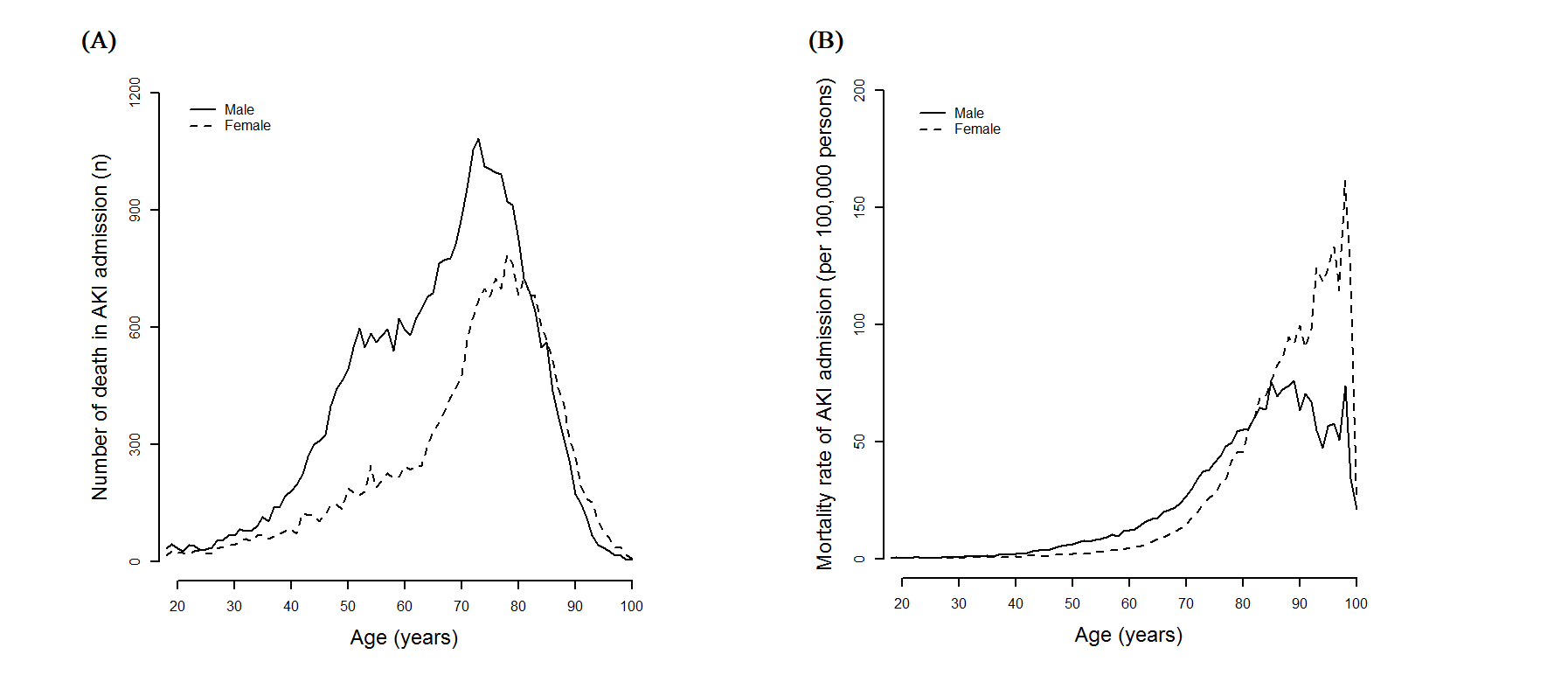

Supplement: Supplementary file 3 — Additional file 3: Fig S2. Number of deaths in critically ill patients with acute kidney injury and age-standardized in-hospital mortality by age and gender in Korea between 2008 and 2015. In-hospital mortality showed an upward trend by age. The increase was more rapid in females than in males after age 70. [file 13613_2019_534_MOESM3_ESM.tif]
